# Efficacy of laser therapy on primary burning mouth syndrome: a systematic review

**DOI:** 10.22514/jofph.2024.003

**Published:** 2024-03-12

**Authors:** Mehdi Khemiss, Nouha Dammak, Oumaima Lajili, Sinda Yacoub, Mohamed Ben Khelifa

**Affiliations:** ^1^Department of Dental Medicine, Fattouma Bourguiba University Hospital of Monastir, 5000 Monastir, Tunisia; ^2^Faculty of Dental Medicine, University of Monastir, 5000 Monastir, Tunisia; ^3^Research Laboratory LR12SP10: Functional and Aesthetic Rehabilitation of the maxilla, Farhat HACHED University Hospital of Sousse, 4000 Sousse, Tunisia

**Keywords:** Pain, Burning mouth syndrome, Stomatodynia, Trigeminal, Photobiomodulation

## Abstract

The objective of this study was to perform a systematic review of the literature to determine the overall efficacy of low-level laser therapy (LLLT) in managing burning mouth syndrome (BMS). A literature search was conducted through PubMed, Scopus, Web of Sciences, and Cochrane Central Register of Controlled Trials from their inception up to 28 March 2023. The search terms were defined by combining (Mesh Terms OR Key Words) from “Burning mouth syndrome” AND (Mesh Terms OR Key Words) from “Laser therapy”. Methodological quality assessment was performed using the Joanna Briggs Institute Critical appraisal tool, attributing scores from 1 to 13 to the selected studies. Literature search, study selection, and data extraction were carried out by two authors. Differences on issues were resolved by a third author, if required. The primary investigated outcome was reducing BMS pain. A total of 21 articles met the inclusion criteria. After assessing full-text articles for eligibility, 12 articles were excluded. Consequently, 9 articles were retained. A low score of bias was calculated in 66% of the included studies. Compared to placebo, a significant reduction in pain or burning sensation was reported in 5 studies. This significant reduction was still observed in the laser group at the two- and four-month follow-ups in 2 studies. LLLT could be beneficial for patients suffering from BMS. In order to get strong evidence for placebo use, future studies with standardized methodology and outcomes are required.

## 1. Introduction

Laser (Light Amplification by Stimulated Emission of Radiation) is a device that creates light energy by the amplification of electromagnetic radiations [1]. It can be used as a tool to cut metal or to create patterns of light entertainment [2]. Because its beam is so small and precise, laser has been used in the medical field. Indeed, it allows health care providers to safely treat tissues without injuring the surrounding area [3]. Laser can be used to treat varicose veins, to improve vision during eye surgery on the cornea, to repair a detached retina of the eye, to remove the prostate, to remove kidney stones, and to remove tumors [3]. Lasers are also useful for esthetic purposes, such as the removal of benign pigmented lesions, hair removal, and tattoo removal [3]. For these reasons, low level laser therapy (LLLT) has emerged as a possible alternative to standard care treatments [4]. However, its effectiveness was not proven and its mechanism of action was not well elucidated [1]. It was only since the beginning of the 21st century that attitude toward LLLT has changed [5].

Lasers have also been used for many dental purposes [6]. The first paper investigating the use of laser in dentistry was published in 1971 by Weichman and Johnson [7], reporting the *in vitro* apical foramen sealing with a high-power carbon dioxide (CO_2_) laser. In dental medicine, lasers may be classified depending on the laser active medium or according to the applicability in hard and soft tissue [8]. Nowadays, lasers have a wide range of applications. High-power lasers, such as Neodymium-doped Yttrium Aluminium Garnet (NdYAG), diodes, and Erbium-doped Yttrium Aluminium Garnet (ErYAG) are used for cutting dental hard tissues, hemostatic ablation of soft tissues, and disinfection of root canals in endodontic treatment [8, 9]. Non-surgical laser using LLLT can help in tissue regeneration, detecting caries, bleaching, curing off composite restoration materials, modulation of inflammation, and management of orofacial pain [8, 10].

LLLT can reduce chronic pain in many pathologies, such as burning mouth syndrome (BMS). “This syndrome is defined as an idiopathic orofacial pain with intraoral burning or dysaesthesia recurring daily for more than two hours and over more than three months. It has no identifiable causative lesions, and it is manifested with and without somatosensory changes” according to the International Classification of Orofacial Pain, 2020 [11]. There is actually no clear consensus on the exact ethiopathogenesis, often idiopathic, of the BMS [12]. LLLT has a significant effect in reducing pain scores among patients suffering from this syndrome [13]. Results are also maintained for one to four months after ten sessions of LLLT. Therefore, this therapy can be used together with pharmacological and psychological treatment for a better outcome to manage BMS. This technique is non-invasive and it does not have any known side effects [13]. Several systematic reviews [4, 14, 15, 16, 17, 18] have suggested that LLLT may be an effective alternative in the treatment of BMS. However, the studies included in these systematic reviews have shown discrepant results, which could be explained by the heterogeneity of the laser parameters already configured, the intervention applied in the control group (placebo or drug), and the assessment tools used. Therefore, more evidence is still required to ensure the efficiency of LLLT in the management of BMS.

A systematic review of the literature was therefore conducted to determine the overall efficiency of LLLT in patients with primary BMS.

## 2. Materials and methods

### 2.1 Protocol and eligibility criteria

The systematic review was conducted in accordance with the guidelines provided in the “Preferred Reporting Items for Systematic Reviews and Meta-Analyses” (PRISMA) guidelines [19]. The review protocol was registered under the “International Prospective Register of Systematic Reviews” (CRD42023402297). The inclusion criteria were specifically constructed following the PICOS criteria: P (population) = patients with primary BMS; I (intervention/exposure) = LLLT; C (Comparison) = patients with primary BMS treated with LLLT versus placebo; O (Outcome) = decrease in pain and burning sensation; and S (Study design) = clinical trials written in English. The review question was “Does the laser have an impact on reducing the pain of patients diagnosed with primary BMS?”.

No restrictions were applied in terms of setting, country or period. Publications not in compliance with the purpose of this systematic review as well as those not representing original research (*i.e.*, reviews, editorials, qualitative papers, case reports, case series, and letters to editors) were excluded.

### 2.2 Search strategy

The search in electronic databases (PubMed, Scopus, Web of Sciences, and Cochrane Central Register of Controlled Trials) was carried out individually by only two investigators (MK and OL in the authors’ list). All databases were consulted looking for the following two “Medical Subject Headings” (MeSH) terms OR Key words: “Burning Mouth Syndrome” OR “Stomatodynia” AND “Laser Therapy”. Articles published until 28 March 2023 were explored. In addition, the reference lists of the included articles were checked.

### 2.3 Study selection

The EndNote™ software (Clarivate, London, UK), v. 9.0 was used to remove duplicates. It was also utilized for the initial screening of the articles on the basis of their title and abstract. Eligible articles meeting the inclusion criteria were further assessed by accessing their full text from relevant sources. This was performed individually by two investigators (MK and OL in the authors’ list). Any discrepancy between the two investigators was resolved by a third investigator (ND in the authors’ list).

### 2.4 Data extraction

Data from the retained studies were extracted using a format including the population, the parameters being investigated, the periods during which the parameters were collected, and the significant findings. Data were extracted, reviewed, and analyzed by two authors (MK and OL in the authors’ list). The extracted data were then verified by two other authors (ND and SY in the authors’ list). Discrepancies in data collection were resolved through discussion.

### 2.5 Methodological quality assessment

Assessment of the risk of bias for all the included studies was carried out using the Joanna Briggs Institute (JBI) critical appraisal tool, precisely the checklist for clinical trials (https://joannabriggs.org). According to the JBI critical appraisal tool, 13 items were taken into consideration to evaluate each article in terms of risk of bias: randomization component, allocation concealment, treatment group similar at the baseline, blinding of participants, blinding of personnel, blinding of outcome assessors, groups treated identically other than the intervention of interest, follow-up, intention to treat, similar way of outcome measurement, reliable way of outcome measurement, statistical analysis and trial design. These items are scored as either yes, no, unclear or not applicable. Two authors (MK and OL in the authors’ list) independently scored the retained studies, with discrepancies resolved through discussion. In case of discrepancy, a third author (ND in the authors’ list) intervened to reach consensus. The risk of bias in the studies was judged to be low (“yes” scores >70%), moderate (“yes” scores between 50% and 69%), and high (“yes” scores <49%) [20].

## 3. Results

### 3.1 Search results

The initial search identified a total of 368 relevant articles from the electronic databases. After the removal of duplicates, only 223 articles were kept. A total of 202 articles were excluded based on the title and the abstract. After assessing full-text articles for eligibility, 12 were excluded for different reasons [2, 21, 22, 23, 24, 25, 26, 27, 28, 29, 30, 31]. At the end of the study selection, 9 articles were included in the present systematic review [32, 33, 34, 35, 36, 37, 38, 39, 40]. The study screening and selection of articles are explained in Fig. 1.

**Fig. 1.** Study flow chart.

### 3.2 Study characteristics

The retained studies were assessed for methodological quality (Table 1). A total of six studies included in this systematic review had a low score of bias [32, 33, 34, 36, 37, 38]. Three other studies had a moderate score of bias [35, 39, 40]. The final scores ranged from 54% to 92%.

**Table 1.** Quality scoring of the retained articles according to JBI critical appraisal checklist.

Author & year	1	2	3	4	5	6	7	8	9	10	11	12	13	Score	Risk of bias
Sun *et al*. [40] 2021	Y	Y	U	Y	N	N	Y	N	U	Y	Y	Y	Y	8	Moderate
De Pedro *et al*. [38] 2020	Y	Y	Y	Y	U	U	Y	Y	Y	Y	Y	Y	Y	11	Low
Scardina *et al*. [33] 2020	Y	Y	Y	Y	N	Y	Y	Y	Y	Y	N	Y	Y	12	Low
Škrinjar *et al*. [37] 2020	Y	Y	Y	Y	N	Y	Y	Y	Y	Y	Y	Y	Y	12	Low
Spanemberg *et al*. [34] 2019	Y	U	Y	Y	N	Y	U	Y	Y	Y	Y	Y	Y	10	Low
Sikora *et al*. [35] 2018	Y	Y	Y	Y	N	N	U	N	Y	Y	Y	Y	Y	9	Moderate
Valenzuela and Lopez-Jornet [32], 2017	Y	Y	Y	Y	U	U	Y	Y	Y	Y	Y	Y	Y	11	Low
Suguaya *et al*. [36] 2016	Y	Y	Y	Y	N	Y	Y	Y	Y	Y	Y	Y	Y	12	Low
Spanemberg *et al*. [39] 2015	Y	Y	U	U	U	U	Y	F	U	Y	Y	Y	Y	7	Moderate

*N: No; NA: Not applied; U: Unclear; Y: Yes.*

Table 2 exposes the main characteristics and the methodology points of the retained studies. The latter were published between 2015 [39] and 2021 [40]. They were conducted in Spain [32, 34, 38], Croatia [35, 37], Brazil [36, 39], and China [40]. The place where the study was carried out was not reported in one study [33]. The number of treated participants varied from 23 [37] to 78 [39], with a wide age range extending from 19 [40] to 88 [32] years. All participants with BMS were appropriately defined as having chronic pain for more than three, four or six months, with normal oral mucosa.

**Table 2.** Characteristics of the main published studies aiming to evaluate the low laser effect in Burning Mouth Syndrome.

Author & year	Location (Country)	Study design	Patients (n, M/F, age)	Period	Inclusion criteria	Non-inclusion criteria	Treatment	Placebo	Duration (Follow-up)	Pain scale	Life quality
Sun *et al*. [40] 2021	Shanghai (China)	Single blinded RCT	42, 34/8, 51.7 (19–71)^b^	2018–2019	Oral burning sensation No detectable organic lesion in the oral cavity Symptom of burning sensation only on the tongue Ability to complete the clinical trial	Patients with a history of psychosis Pregnancy or breast-feeding women	Laser treatment	Silent/off laser therapy	4 weeks (NR)	VAS	NR
De Pedro *et al*. [38] 2020	Madrid (Spain)	Single blinded RCT	TG: 20, 2/8 60.30 ± 15.19^a^ CG: 20, 2/8 67.60 ± 10.68^a^	2019	age ˃18 years Diagnosis of BMS	Hyposalivation or Sjögren’s syndrome Antecedent of head and neck radiotherapy Pregnant women Patients with uncontrolled systemic diseases Patients suffering from burning mouth symptoms secondary to local factors	Laser treatment	Silent/off laser therapy	5 weeks (4 months)	VAS MPQ	OHIP-14
Scardina *et al*. [33] 2020	NR (NR)	Double blinded RCT	40, 0/40, 62.06 ± 3.1^a^	NR	Diagnosis of BMS Patients who had healthy mucosa	Candidiasis, lichen planus, glossitis, periodontitis Systemic pathologies Smokers Previous appearance of mycosis Hypertension Patients with daily pharmacological treatments	Laser treatment	Silent/off laser therapy	4 weeks (60 days)	VAS NRS	NR
Škrinjar *et al*. [37] 2020	Zagreb (Croatia)	Double blinded RCT	TG: 12, 1/11, 61 (47–70)^b^ CG: 11, 2/9, 62 (50–69)^b^	NR	Burning (˃3 months) Normal appearance of the oral mucosa	Diabetes Serum iron and vitamin B deficiency Previous head and neck radiotherapy Patients with autoimmune diseases Patients taking antidepressants, anxiolytics, anticonvulsants and hormonal therapy	Laser treatment	Silent/off laser therapy	10 days (NR)	VAS	NR
Spanemberg *et al*. [34] 2019	Barcelona (Spain)	Double blinded RCT	21, 1/20, 66.3 ± 6.9^a^ (61–81)^c^ TG: 12, 66.3 ± 7.5^a^ CG: 9, 66.4 ± 6.3^a^	NR	Patients over 40 years old Burning or pain in the oral mucosa of at least 3 months of duration	Patients with uncontrolled systemic diseases Patients without clinical activity of BMS, or a VAS score below 3	Laser treatment	Silent/off laser therapy	4 weeks (2 months)	VAS	NR
Sikora *et al*. [35] 2018	Osijek (Croatia)	Single blinded RCT	44, 1/43, 67.6 (56–83)^b^	2015	Burning sensation Clinically normal oral mucosa Absence of local and systemic factors that can lead to burning sensation of the oral mucosa	Inability to comprehend the text of the informed consent form and inability to comprehend the questionnaire	Laser treatment	Silent/off laser therapy	14 days (NR)	VAS	OHIP-14
Valenzuela and Lopez-Jornet [32], 2017	Murcia (Spain)	RCT	44, 3/41, 65.5 ± 10.6^a^ (33–88)^c^	NR	Diagnosis of BMS Patients with continuous burning/pain on a daily or almost daily basis during all/part of the day for more than 6 months No local/systemic factors that could produce the same symptoms	A history of head and neck malignancy radiation Diabetes mellitus Chronic thyroid disease Known Sjögren’s syndrome Fibromyalgia and rheumatoid arthritis Anemia Analgesic and/or anti-inflammatory medications Pregnancy Unwillingness to give consent to participate	Laser treatment	Silent/off laser therapy	4 weeks (NR)	VAS	OHIP-14
Suguaya *et al*. [36] 2016	Sao Paulo (Brazil)	Double blinded RCT	23, 2/21, 59.7 (29–83)^b^ TG: 13, 0/13, 57.3 (29–83)^b^ CG: 10, 2/8, 62.7 (53–81)^b^	NR	Patients meeting the diagnostic criteria for BMS	Clinical alterations in the oral mucosa Hyposalivation Diabetes B hypovitaminosis Anemia Bearing in mind the laser radiation Previous malignant/benign head + neck neoplasia Pregnant and breastfeeding women	Laser treatment	Silent/off laser therapy	2 weeks (90 days)	VAS	NR
Spanemberg *et al*. [39] 2015	Rio (Brazil)	RCT	78 (67/11) TG_1_: 20, 3/17, 63.6 ± 9.6^a^ TG_2_: 20, 2/18, 60.5 ± 6.4^a^ TG_3_: 19, 1/18, 63.2 ± 6.9^a^ CG: 19, 5/14, 61.5 ± 8.8^a^	NR	Burning or pain in the oral mucosa for at least 6 months A clinically normal mucosa	Antidepressant, anxiolytic, or anticonvulsant medication Previous chemo- and/or radiotherapy Salivary flow rate at rest ≤0.1 mL/min Alterations in their blood count, glucose serum levels, iron, folic acid and vitamin B12	Laser treatment	Silent/off laser therapy	TG_1_: 10 weeks (8 weeks) TG_2_, TG_3_, CG: 3 weeks (8 weeks)	VAS	OHIP-14

*CG: Control group; CT: Controlled trial; F: Female; M: Male; MPQ: Mc Gill Questionnaire; NR: Nor reported; NRS: Numeric rating scale; OHIP: Oral health impact profile; RCT: Randomized controlled trial; TG: Treatment group; VAS: Visual analogic scale; Data were: ^a^Mean ± standard deviation, ^b^Median (Minimum–maximum), ^c^Minimum–maximum.*

Randomization was applied in nine studies [32, 33, 34, 35, 36, 37, 38, 39, 40] (Table 1). All the included studies [32, 33, 34, 35, 36, 37, 38, 39, 40] were controlled clinical trials, of which four were double-blinded studies [33, 34, 36, 37] (Table 1). All the studies [32, 33, 34, 35, 36, 37, 38, 39, 40] reported data regarding items 10 (*i.e.*, similar way of outcome measurement) and 12 (*i.e.*, statistical analysis) (Table 1). Nine studies [32, 33, 34, 35, 36, 37, 38, 39, 40] with treatment duration varying between ten days and five weeks were categorized as short-term assessment. The number of sessions varied between once a week and three times a week (Table 3). At the end of the intervention, follow-up was reported in six studies [33, 34, 36, 38, 39, 40], ranging between one week and four months (Table 2).

### 3.3 Laser characteristics

LLLT modalities are summarized in Table 3. Diode laser was used in nine studies [32, 33, 34, 35, 36, 37, 38, 39], with wavelengths and power ranging from 685 [37, 39] to 830 nanometers (nm) [35, 39] and from one [32] to 4000 microwaves (mW) [33]. Sun *et al*. [40] applied NdYAG laser. In seven studies [32, 35, 36, 37, 38, 39, 40], energy fluence and irradiation area of laser were reported, varying from three [40] to 200 [32] joules per square centimeter (J/cm^2^) and from 0.028 [39] to three cm^2^ [37], respectively. All the studies [32, 33, 34, 35, 36, 37, 38, 39, 40] reported a number of applications varying from four [33] to 56 [38], with an exposure time per point between four [32] and 381 seconds [37]. Participants in six studies received constant sessions [32, 33, 34, 36, 38, 39]. Only Sun *et al*. [40] applied the pulsed mode. The distance between the probe and the irradiated area was reported in three studies [33, 37, 40].

Placebo was administrated in the same way as the active treatment in all the studies. Silent/off laser therapy in contact with the mucosa was applied as a placebo treatment in all the retained studies [32, 33, 34, 35, 36, 37, 38, 39, 40] (Table 2).

The following anatomic sites were chosen as points where LLLT was applied: tongue, buccal mucosa, lips, hard palate, soft palate, and alveolar ridge mucosa (Table 3).

**Table 3.** Photobiomodulation characteristics of the main published studies.

Author & year	Laser Feature	Wave length (nm)	Power (mW)	Energy fluence (J/cm^2^)	Irradiation area (cm^2^)	Exposure time/point (S)	Number of session (duration)	Mode	Number of applications	Localization	Laser distance (cm)
Sun *et al*. [40] 2021	Nd:YAG	1064	100	3	1	30	One per week (4 weeks)	Pulsed	17	Tongue	0.6
De Pedro *et al*. [38] 2020	Diode laser fox	810	60	12	0.5	6	Twice a week (5 weeks)	Continuous	56	Vestibular mucosa, lip mucosa, buccal mucosa, hard palate, tongue	NR
Scardina *et al*. [33] 2020	Diode led	800	4000	50	NR	300	Twice a week (4 weeks)	Continuous	4	Upper labial mucosa, lower labial mucosa, buccal mucosa, dorsal tongue	4
Škrinjar *et al*. [37] 2020	Ga Al As LED laser light	685	30	60	3	381	One per day (10 days)	NR	10	NR	0.5
Spanemberg *et al*. [34] 2019	Thor laser gallium and aluminum arsenide diode laser	808	200	NR	0.088	15	Twice a week (4 weeks)	Continuous	44	Tongue, buccal mucosa, labial mucosa, hard palate, soft palate, gingival or alveolar mucosa	NR
Sikora *et al*. [35] 2018	Ga AL As Laser	830	100	12	1	300	One per day (10 days)	NR	10	NR	NR
Valenzuela and Lopez-Jornet [32], 2017	Ga Al As Laser	815	1	133.3 200	0.03	4 6	NR (4 weeks)	Continuous	10	NR	NR
Suguaya *et al*. [36] 2016	Infrared diode laser	790	120	6	0.03	50	NR (2 weeks)	Continuous	TG: 24 CG: 17	Tongue, lower lip, upper lip, buccal mucosa, mandibular ridge, palate gingiva, mandibular gingiva	NR
Spanemberg *et al*. [39] 2015	Infrared diode laser Red diode laser	830 685	100 35	176 72	0.028	50 58	One per week (10 weeks) 3 times a week (9 weeks)	Continuous	44	Tongue, buccal mucosa, labial mucosa, hard palate, soft palate, gums, alveolar ridge mucosa	NR

*CG: Control group; J/cm^2^: joules per square centimeter; mW: microwave; nm: nanometer; Nd:YAG: neodymium-doped yttrium aluminium garnet; NR: Nor reported; TG: Treatment group; S: second.*

### 3.4 Outcome assessment

Pain and/or burning intensity evaluation was the primary outcome of all the included studies [32, 33, 34, 35, 36, 37, 38, 39, 40]. The Visual Analog Scale (VAS) was the principal assessment tool used in measuring pain intensity. It was used in nine studies [32, 33, 34, 35, 36, 37, 38, 39, 40]. The pain intensity was measured differently in one of the aforementioned studies. The VAS values were presented by percentages [36]. Supplementary assessment tools, such as the Mc Gill Pain Questionnaire (MPQ) [38] and the Numeric Rating Scale (NRS) [33, 39] were also used to evaluate pain (Table 2).

Participants’ secondary outcome assessment, such as the quality of health, anxiety, depression, and the quality of sleep were evaluated using patient-reported questionnaires, including OHIP-14 [32, 35, 38, 39], hospital anxiety depression [32, 34], and xerostomia inventory [32].

### 3.5 Laser effects on burning mouth syndrome

A total of five studies [32, 34, 38, 39, 40] reported a significant reduction in pain or burning sensation compared to placebo (Table 4). This significant reduction was still observed in the laser group at the two- and the four-month follow-ups [38, 39]. Pain was notably reduced in both laser and placebo groups [33, 35, 36, 37] in four studies. After two months, the recurrence of burning sensation in the control group was reported in one study [33].

LLLT was effective in improving the quality of life in one study [32]. However, de Pedro *et al*. [38] reported an irrelevant result between the laser group and the control group (Table 4). Valenzuela and Lopez-Jornet [32] did not report any significant change in both xerostomia severity and hospital anxiety depression in the laser group or the control group.

**Table 4.** Effects of photobiomodulation in pain/burning and quality of life.

Author, year	Data	Baseline	End of the treatment	End of the follow up	Treatment Vs Placebo	Conclusion
Sun *et al*. [40] 2021	VAS^a^	TG = 4.5 ± 1.7 CG = 4.1 ± 1.5	TG = 2.2 ± 1.1^*^ CG = 3.6 ± 1.4^*^	NR	A significant difference of VAS in pain/burning was observed between TG and CR at the end of the treatment	LLLT was effective for reduction of pain in patients with BMS
De Pedro *et al*. [38] 2020	VAS^b^	TG = 6.8 CG = 7.1	TG = 3.4^*^ CG = 7.6	TG = 3.9^†^ CG = 7.6	VAS for pain decreased significantly in the TG *vs.* the CG at the end of treatment and 4-month follow-up	LLLT was effective for reduction of pain in patients with BMS
	OHIP-14^a^	TG = 16.5 ± 13.1 CG = 21.3 ± 9.1	TG = 14.4 ± 8.2 CG = 22.0 ± 8.4	TG = 12.5 ± 7.1 CG = 24.1 ± 11.5	No significant differences were found between the TG and the CR	
Scardina *et al*. [33] 2020	NRS^b^	TG = 7 CG = 7	TG = 3^*^ CG = 5^*^	TG = 3^†^ CG = 7	Improvement was seen on the NRS of the linear type in the 2 groups After 2 months, patients in CG showed a recurrence of burning sensation	LLLT decreased pain symptoms in patients with BMS but no difference compared to placebo
Škrinjar *et al*. [37] 2020	VAS^c^	TG: 5.5 (4–9) CG: 5 (0–8)	TG: 4 (3–7)^*^ CG : 3 (1.5–6.5)^*^	NR	The results have shown that VAS scores were significantly lower in both groups	LLLT decreased pain symptoms in patients with BMS but no difference compared to placebo
Spanemberg *et al*. [34] 2019	VAS^b^	TG = 8.9 CG = 8.3	TG = 5.5^*^ CG = 5.8^*^	TG = 4.7^†^ CG = 5.1^†^	Clear significance in the improvement of pain for the TG compared to the CG the two-month period and at the end of the follow up	LLLT was effective for reduction of pain in patients with BMS
Sikora *et al*. [35] 2018	Δ_VAS_^a^	TG = 1.4 ± 3.3^*^ CG = 2.4 ± 2.9^*^		NR	NR	LLLT decreased pain symptoms in patients with BMS but its effectiveness cannot be given since the authors did not make a comparison between the test group and the control group
	Δ_OHIP-14_^a^	TG = 2.7 ± 8.6 CG = 1.3 ± 5.6				
Valenzuela and Lopez-Jornet [32], 2017	VAS^a^	TG = 7.6 ± 1.5 TG’ = 8.4 ± 1.7 CG = 7.8 ± 1.3	TG = 6.4 ± 1.6^*^ TG’ = 7.1 ± 1.8^*^ CG = 7.6 ± 1.2	NR	VAS scores obtained from the two groups treated with laser were significantly lower than scores from placebo group	LLLT was effective for reduction of pain in patients with BMS
	OHIP-14^a^	TG = 29.9 ± 3.6 TG’ = 29.6 ± 5.9 CG = 29.3 ± 5.9	TG = 28.5 ± 3.1^*^ TG’ = 28.2 ± 6.1^*^ CG = 29.2 ± 6.3	NR	OHIP-14 scores among patients treated with LLLT showed significant decreases from baseline to 2 weeks treatment	
Suguaya *et al*. [36] 2016	n	TG: Six of the 13 patients reported complete remission of symptoms in all sites affected by the burning sensation at the last control checkpoint. CG: Four of the 10 patients reported total remission of symptoms in all affected sites at the end of the control period.			The laser protocol used to treat this group of BMS patients produced benefits similar to those of the placebo treatment applied	LLLT decreased pain symptoms in patients with BMS but no difference compared to placebo
Spanemberg *et al*. [39] 2015	NRS^a^	TG = 8.2 ± 1.6 TG’ = 8.0 ± 1.3 TG” = 8.2 ± 1.7 CG = 9.0 ± 1.0	TG = 3.2 ± 2.5^*^ TG’ = 3.0 ± 2.3^*^ TG” = 4.3 ± 2.7^*^ CG = 6.0 ± 1.2^*^	TG = 3.7 ± 2.4^†^ TG’ = 2.9 ± 2.1^†^ TG” = 4.4 ± 2.7^†^ CG = 6.5 ± 2.3^†^	NRS scores obtained from TR and TR’ treated with Infrared laser were significantly lower than scores from control group	LLLT was effective for reduction of pain in patients with BMS
	VAS^a^	TG = 82.1 ± 14.5 TG’ = 78.9 ± 15.2 TG” = 80.7 ± 18.6 CG = 85.7 ± 14.2	TG = 28.2 ± 27.2^*^ TG’ = 30.8 ± 24.1^*^ TG” = 44.9 ± 28.3^*^ CG = 66.4 ± 19.8^*^	TG = 32.9 ± 28.9^†^ TG’ = 25.9 ± 19.5^†^ TG” = 41.1 ± 27.1^†^ CG = 62.8 ± 26.3^†^	VAS scores obtained from TR’ treated with one infrared laser were significantly lower than scores from control group	
	OHIP-14^a^	TG = 13.8 ± 7.5 TG’ = 12.9 ± 7.8 TG” = 14.7 ± 7.2 CG = 17.8 ± 5.4	TG = 8.5 ± 5.1^*^ TG’ = 6.9 ± 4.1^*^ TG” = 9.8 ± 4.9^*^ CG = 13.39 ± 3.6^*^	NR	OHIP-14 scores obtained from TR’ treated with one infrared laser were significantly lower than scores from control group	

BMS: Burning mouth syndrome; CG: Control group; LLLT: Low level laser therapy; n: number; NR: Nor reported; NRS: Numeric rating scale; OHIP: Oral health impact profile; TG: Treatment group; VAS: Visual analogic scale. Data were: ^a^Mean ± standard deviation, ^b^Mean, ^c^Median (Minimum–maximum); ^*^p < 0.05 (End of the treatment vs. Baseline), ^†^p < 0.05 (End of the follow-up vs. Baseline).

## 4. Discussion

The present systematic review included nine clinical trials and it aimed to evaluate the efficacy of LLLT in the treatment of BMS. Photobiomodulation was found to reduce pain and burning among patients suffering from this syndrome. However, laser parameters used during the therapy varied widely in the included studies. It is therefore difficult to provide a clear conclusion.

### 4.1 Scope of the study

BMS is an idiopathic orofacial pain which affects patients’ life quality. It also has a negative impact on patients’ emotions. This situation can worsen to the point that “patients are suspected of imagining or exaggerating their symptoms” [41]. Actually, there is a lack of robust scientific evidence in the treatment modalities proposed to manage BMS [12, 13]. Consequently, the management options based on patients’ symptomatology often leads to unsatisfactory results. Reyad *et al*. [42] conducted a systematic review evaluating the effectiveness of both pharmacological and non-pharmacological treatments during BMS. The authors concluded that there is no consensus on how to treat this chronic disorder [42]. The treatment success depends on the following number of issues [43]: correct diagnosis, confirmation of diagnosis, patient’s acceptance, patient’s understanding of the likely clinical course, patient’s participation in the elaboration of a treatment strategy, patient’s non-compliance, positive feedback during treatment, ongoing interest of the clinicians, and the side effects of the drugs. LLLT can therefore be of great use in reducing burning and the associated symptoms in patients diagnosed with BMS.

### 4.2 Efficacy of laser therapy

Laser analgesia is a non-invasive, non-destructive, and non-thermal biomodulation method that reduces or eliminates pain sensation. It is obtained by a low-energy-level irradiation form [44]. This systematic review revealed that LLLT may be efficient in reducing pain caused by BMS. In addition, the studies included in this systematic review reported a short-term assessment consolidating the LLLT analgesic effect. The reduction in symptoms was still evident four months later despite the end of the intervention in one study [38]. This finding is corroborated by the successful use of photobiomodulation in reducing pain during many diseases, such as recurrent aphthous stomatitis and oral lichen planus [45], temporomandibular joint disorders [46], oral mucositis induced by radiation therapy [47] …

However, no study has evaluated if LLLT is able to reduce pain/burning with a constant and long-lasting effect. Sugaya *et al*. [36] suggested that the effect of LLLT lasts longer when laser irradiation is applied in several sessions compared to a single application. Therefore, studies with a long-term follow-up are required to assess the lasting effect of this alternative treatment. This may explain the non-effectiveness of LLLT in life quality improvement among patients suffering from BMS. Indeed, when assessing the impact of BMS, it is important to take into account the oral health-related quality of life. The latter was evaluated using OHIP-14 in four studies [32, 35, 38, 39] included in the present review. Only one study found that OHIP-14 scores decreased among the laser group from baseline to two weeks treatment compared to the control group [32].

The studies included in the present systematic review did not report any side effects. Therefore, photobiomodulation may be considered as a safe application in the treatment of BMS [48]. However, potential adverse events still need to be investigated. In addition, laser beams must not aim at the eyes and both patients and operators should wear wavelength-appropriate safety spectacles [48].

### 4.3 Biological mechanisms underlying the laser therapy effect

Although many theories have been suggested to explain laser analgesic effect, the causes of this effect are not totally clear [44]. It seems to be consecutive to photobiomodulation produced by all laser when emitted with proper parameters [49]. This term is nowadays used to describe the wide range of LLLT [49]. “Photobiomodulation” was added as a MeSH term in the National Library of Medicine’s controlled vocabulary thesaurus in 2015 [50]. Since then, it has taken the place of terms such as “biostimulation”, “soft laser” or “cold laser” [49]. Many mechanisms are involved in producing photobiomodulation. The most accepted theory is that in the mitochondria, the photon energy is absorbed by cytochrome C oxidase (CcOx), a mitochondrial chromophore localized in the cellular membrane. This absorption leads to an increase in adenosine triphosphate (ATP) and modulation of reactive oxygen species (ROS), followed by a diminution of oxidative stress. In these stressed cells, the oxygen is replaced by nitric oxide (NO) that competitively binds to CcOx. This reaction inhibits cellular respiration and ATP production [16]. The intracellular release of Ca^2+^ is another possible mechanism that may explain the effect of photobiomodulation. Indeed, cellular Ca^2+^ concentration regulates some reactions and it is essential for signal transduction [16]. The activation of different transcription factors and the stimulation of signaling pathways lead to cellular proliferation and the production of antioxidant factors, anti-inflammatory proangiogenic factors, and anti-apoptotic activities [4]. Indeed, photobiomodulation modifies nerve conduction and peripheral neurons excitability using its action on the Na^+^/K^+^ pump, which explains the inhibition of nociceptive stimulation [4]. Finally, the relief in burning sensation may be due to the liberation of ß-endorphins and enkephalins, which are natural painkillers [14]. It can also be explained by the secretion of pain mediators, such as bradykinin and histamine [14]. Although more investigations are necessary to clarify the biological mechanisms of photobiomodulation, it is possible to conclude that a small stimulus of LLLT may be without biological effect while a large stimulus can cause inhibitory or cytotoxic effects [15]. In addition, it is important to point out that the laser analgesic effect is linked to the etiopathology of the BMS. In fact, some patients suffering from this syndrome have a central pain that may include hypofunction of dopaminergic neurons in the basal ganglia [51]. Therefore, LLLT which is topical, will probably not be useful.

### 4.4 Discussion of the methodology

RCTs are widely considered as the most rigorous method for evaluating the treatment efficacy or preventive interventions [52]. In fact, 66% of the included studies had a low score of risk of bias. It is known that systematic reviews may be affected by bias at the level of individual studies [53]. For this reason, an assessment of the validity of these studies is a crucial step when conducting a systematic review [54]. Indeed, the true intervention effect may be overestimated or underestimated [53].

In the included studies, pain/burning in the oral mucosa was the patients’ principal outcome assessment. It was evaluated using many assessment tools. VAS was used in nine studies [32, 33, 34, 35, 36, 37, 38, 39, 40]. It is a continuous and a uni-dimensional pain rating scale widely used in diverse adult populations [38].

OHIP-14 was used by some authors to assess the impact of BMS on patients’ life. It was originally developed by Slade and Spencer [55] in Australia. It contains items related to seven dimensions: functional limitation, physical pain, psychological discomfort, physical disability, psychological disability, social disability and handicap [55]. It is the most widely used both by researchers and clinicians [56].

### 4.5 Study limitations

The current systematic review presents five limitations. The first one is related to the high variability in laser delivery parameters used during the therapy including the wavelength, power, energy fluence dose, irradiation area, exposure time/point, number of sessions, and points of application (Table 3). This might be the reason for the discrepancy in the results between the included studies [16]. It is therefore necessary to determine the optimum treatment frequency and dose which “represents the total amount of energy delivered to the surface unit area” [16]. Current data show that higher doses of light have positive effects in relieving pain compared to small doses which are not beneficial. However, excess energy may lead to photobioinhibition rather than photobiostimulation [16]. Based on the results of this review, the following laser delivery parameters may be applied twice a week during one month at least when managing patients with BMS: wavelength between 800 and 830 nm, power between 60 and 200 mW, irradiation area <1 cm^2^, and exposure time per pulsion <1 min.

Another reason which may explain the discrepancy in the results between the included studies, is the heterogeneity of the inclusion criteria applied by the authors in the nine studies. This heterogeneity may be considered as a second limitation of the present review. The third limitation concerns the number of included patients in the nine studies, which varied between 23 and 78. Indeed, no study has reported “sample size calculation”. With regard to this issue, future studies should include a proper sample size since an optimal size is a crucial point to avoid an adequate power in order to detect statistical effects [57]. The fourth limitation is related to the duration of the therapy. Patients were followed-up for a short period of time whereas the pain occurring in BMS is chronic. Future studies should last more than four months. The fifth limitation concerns the assessment of pain and/or burning intensity. In fact, tools were applied in different ways.

## 5. Conclusion

Laser therapy may sometimes be both beneficial and ethically acceptable. The LLLT effect, found in this systematic review, represents a significant challenge for future RCTs evaluating therapies for BMS. In order to obtain strong evidence for laser use, a standard protocol should be respected. An adequately long follow-up period must be established to discern if the treatment is more effective than the placebo.

## 6. Highlights

• Key finding: Laser therapy may be efficient in reducing pain caused by burning mouth syndrome;

• Clinical implication: low-level laser therapy can be used as treatment of burning mouth syndrome in some cases especially since there is actually no treatment proven to be a gold standard in the management of this syndrome.
